# Race, Depressive Symptoms, and All-Cause Mortality in the United States

**DOI:** 10.3389/fpubh.2016.00040

**Published:** 2016-03-17

**Authors:** Shervin Assari, Ehsan Moazen-Zadeh, Maryam Moghani Lankarani, Valerie Micol-Foster

**Affiliations:** ^1^Department of Psychiatry, University of Michigan, Ann Arbor, MI, USA; ^2^Center for Research on Ethnicity, Culture and Health, School of Public Health, University of Michigan, Ann Arbor, MI, USA; ^3^Mental Health Research Center, Tehran Psychiatric Institute, School of Behavioral Sciences and Mental Health, Iran University of Medical Sciences, Tehran, Iran

**Keywords:** population groups, ethnic groups, African Americans, depressive symptoms, self-rated health, mortality, chronic medical conditions

## Abstract

**Purpose:**

Despite the well-established association between baseline depressive symptoms and risk of all cause-mortality, limited information exists on racial differences in the residual effects of baseline depressive symptoms above and beyond socioeconomic status (SES) and physical health on this link. The current study compared Blacks and Whites for the residual effects of depressive symptoms over SES and health on risk of long-term all-cause mortality in the U.S.

**Methods:**

Data were obtained from the Americans’ Changing Lives Study, a nationally representative longitudinal cohort of U.S. adults with up to 25 years of follow-up. The study followed 3,361 Blacks and Whites for all-cause mortality between 1986 and 2011. The main predictor of interest was baseline depressive symptoms measured at 1986 using an 11-item Center for Epidemiological Studies-Depression scale. Covariates included baseline demographics (age and gender), SES (education and income), and health [chronic medical conditions (CMCs), self-rated health (SRH), and body mass index (BMI)] measured at 1986. Race (Black versus White) was the focal moderator. We ran a series of Cox proportional hazard models in the pooled sample and also stratified by race.

**Results:**

In the pooled sample, higher depressive symptoms at baseline were associated with higher risk of all-cause mortality except when the CMC, SRH, and BMI were added to the model. In this later model, race interacted with baseline depressive symptoms, suggesting a larger effect of depressive symptoms on mortality among Whites compared to Blacks. Among Whites, depressive symptoms were associated with increased risk of mortality, after controlling for SES but not after controlling for health (CMC, SRH, and BMI). Among Blacks, depressive symptoms were not associated with mortality before health was introduced to the model. After controlling for health, baseline depressive symptoms showed an inverse association with all-cause mortality among Blacks. Although the effect of baseline depressive symptoms on mortality disappeared after controlling for health among Whites, SRH did not interfere (confound) with the effect of depressive symptoms on mortality among Blacks.

**Conclusion:**

The effect of depressive symptoms on increased risk of all-cause mortality, which existed among Whites, could not be found for Blacks. In addition, race may modify the roles that SES and health play regarding the link between depressive symptoms and mortality over a long period of time.

## Introduction

A considerable body of literature has shown that baseline depression and depressive symptoms predict increased risk of subsequent mortality due to all- or specific-causes (1–6). Growing evidence suggests that psychosocial and medical correlates of depression depend on race and ethnicity (7–16). Similar to racial differences in the predictive role of depressive symptoms on long-term mortality (17–21), Blacks and Whites may also differ in the role of baseline depressive symptoms on mortality. In 2015, a study by Assari and Burgard showed that the predictive role of depressive symptoms on renal disease mortality may be limited to Whites and was not found among Blacks (6).

There is a need to study the residual effect of depression or depressive symptoms above and beyond demographics, socioeconomic status (SES), and overall and health indicators such as self-rated health (SRH), chronic medical conditions (CMCs), and body mass index (BMI) (1, 2). As CMC (6, 9, 22–25), SRH (7, 17, 26–28), and BMI (8, 29–34) are all correlated with depression and mortality (6, 9), these health variables potentially confound, mediate, or suppress the predictive role of baseline depressive symptoms on risk of subsequent mortality. However, very few studies have specifically investigated how depressive symptoms operate in conjunction with SES and health status in predicting the long-term risk of mortality.

Race and ethnicity alter the complex associations between SES, depression, and health (7, 8, 17, 26–28, 32–36). Previous research has shown that race and ethnicity alter the pattern of depression and SRH (10) as well as the comorbidity between depression and obesity (7, 8, 32, 33), and depression and CMC (11, 12). Race and ethnicity may also modify the residual effects of SES over health on mortality (18, 20, 28). The race-specific role of depressive symptoms and SRH may be a consequence of racial differences in joint distribution of the risk and protective factors, racial differences in patterns of associations between demographics, SES, physical health, and mental health, or race and ethnic differences in vulnerabilities to the effect of risk and protective factors (6, 17–19). In addition, contextual factors such as race and ethnicity shape the additive effects of SES, depression, and physical health (9, 14, 32, 37–39) even when their separate effects are similar (10, 32, 36, 39). Finally, some of the health indicators may not reflect the same aspects of health across populations (17, 36). Thus, Blacks and Whites may differ in mediating, confounding, or suppressor effects of SES and health (e.g., CMC, SRH, and BMI) on the depressive symptoms – mortality link.

We conducted this study to compare Blacks and Whites for effects of depressive symptoms over SES and health (e.g., CMC, SRH, and BMI) in predicting long-term risk of all-cause mortality in a nationally representative cohort of American adults.

## Materials and Methods

### Design and Setting

Data were obtained from Americans’ Changing Lives (ACL) study (29). The ACL is a nationally representative longitudinal cohort of U.S. adults, who are 25 years or older, followed from 1986 to 2011. More detailed methodology of the study has been previously described (40, 41).

### Sampling and Participants

Stratified multistage probability sampling was applied in the ACL study. At baseline, the study population consisted of 3,617 adults older than 24 years old. Non-institutionalized continental U.S. residents were included in the study. Wave 1 represented 70% of sampled households and 68% of sampled individuals. Blacks and older adults (age >60 years) were oversampled in ACL. We focused on Whites and Blacks in our analysis (total *N* = 3,361, Whites = 2,205, and Blacks = 1,156).

#### Process and Measures

Face-to-face interviews were used to collect baseline data at wave 1.

#### Mortality

The death certificates or the National Death Index (NDI) was used to extract the all-cause mortality data, including the date of death and the cause of death. Death certificates are filled out by physicians after a person’s death in the U.S., and it is used as the source of the NDI. (6, 17)

#### Depressive Symptoms

We used the 11-item Center for Epidemiological Studies-Depression scale (CES-D) (41, 42). In this shortened version of the CES-D, a score of 1–3 is given to each item. We used the mean item score as the total score, ranging from 1 to 3. The abbreviated CES-D has been used in other studies and has shown acceptable reliability and validity (43–45). A cut point (17.84) of mean + SD (14.03 + 3.81) was used to determine the high depressive symptoms at baseline.

#### Chronic Medical Condition

Participants were asked whether they had been informed by a health care provider about having any of the seven focal conditions of interest, including hypertension, diabetes, chronic lung disease, heart disease, stroke, cancer, and arthritis. Answers to each condition were scored as 0 or 1, resulting in a scale of 0–7 for total CMC (9, 41).

#### Self-rated Health

Self-rated health was a single-item scale on subjective overall health, with five categories: poor, fair, good, very good, and excellent. SRH was treated as a dichotomous measure, where the original five categories were collapsed to the poor/fair versus good/very good/excellent categories. SRH has shown acceptable test–retest reliability and validity (26, 45–49).

#### Demographic Factors

Data were collected at baseline on gender (male considered the reference category) and age (in years).

#### Socioeconomic Status

Data were collected at baseline on education (years of schooling) and household income (in continuous scale).

#### Race

Participant’s race was based on self-reported race and ethnicity, collected at baseline in 1986 with several survey items. Participants responded open-endedly to the question, “In addition to being American, what do you think of as your ethnic background or origins?” Respondents were then asked a multiple-choice question, “Are you white, black, American Indian, Asian, or another race?” and allowed to answer with multiple categories. This analysis only included non-Hispanic White and non-Hispanic Black respondents. All Hispanic individuals were dropped from the analysis.

### Ethics

Informed consent was obtained from all participants of the ACL study. Our study was approved by the University of Michigan institutional review board in accordance with The Code of Ethics of the World Medical Association (Declaration of Helsinki, Edinburgh 2000 revision).

### Statistical Analysis

Stata 13.0 (Stata Corp., College Station, TX, USA) was used to account for the complex sample design of the ACL study by applying sampling and non-response weights. Taylor series linearization was applied for the estimation of standard errors in a subsample analysis framework. Hazard ratios (95% confidence interval) were reported. A *P*-value <0.05 was considered significant.

The only main predictor of interest was baseline depressive symptoms measured at 1986. Covariates included baseline SES (education and income), SRH, CMC, and BMI all measured at 1986. Outcome was time to all-cause mortality from 1986 to 2011. Time to all-cause mortality was calculated in months as the timeframe between baseline and death. Time to censoring was calculated in months as the timeframe between baseline and loss to follow-up or baseline and 2011.

A series of Cox proportional hazards models were used to assess the residual effect of baseline depressive symptoms on all-cause mortality over demographics, SES, and health. First, we ran four models in the pooled sample. Model 1 only included high depressive symptoms at baseline (CES-D > mean) and demographic variables. Model 3 was the full model as it controlled for CMC, SRH, and BMI as well as other variables. Model 4 included the interaction term between race and high depressive symptoms (CES-D > mean) at baseline. In the next step, we ran four models for Whites and four models for Blacks in a step-by-step fashion. Model 1 only included depressive symptoms and demographics. Model 2 included socioeconomic variables as well. Model 4 accounted for all the variables.

We used *estat concordance* command in Stata to calculate the *C* statistics for our Cox regression models in the pooled sample based on race. Harrell’s *C* [(*E* + *T*/2)/*P*] was 0.82, 0.84, and 0.79 in the pooled sample, Whites, and Blacks, respectively. Somers’ *D* statistics were 0.64, 0.67, and 0.59 for the pooled sample, Whites, and Blacks, respectively. Thus, performance of the model was only slightly lower among Blacks compared to Whites. Despite reporting *C* statistics, we did not aim to measure overall adequacy of our models, as we did not aim to find risk prediction models. Instead, our aim was to test one association of interest among Blacks and Whites (50–53).

## Results

The total number of deceased and surviving individuals, including both Blacks and Whites, were 1,737 and 1,624, respectively, at the time of our study. Table 1 represents detailed descriptive statistics on all variables included in our study. Whites and Blacks did not differ in age and gender. However, compared to Whites, Blacks had significantly lower SES (education and income), higher depressive symptoms, and poorer health (CMC, BMI, and SRH) at baseline.

**Table 1:** **Descriptive statistics for the analytic sample, stratified by race and overall**.

	Whites		Blacks		All	
	Mean (SE)	95% CI	Mean (SE)	95% CI	Mean (SE)	95% CI
Age	47.96 (0.60)	46.75–49.17	46.33 (0.72)	44.89–47.78	47.77 (0.53)	46.69–48.84
Education*	12.69 (0.11)	12.48–12.90	11.37 (0.23)	10.90–11.84	12.53 (0.10)	12.34–12.73
Income*	5.57 (0.10)	5.36–5.77	4.25 (0.18)	3.88–4.62	5.41 (0.09)	5.22–5.60
Chronic medical conditions*	0.78 (0.03)	0.71–0.84	0.91 (0.05)	0.81–1.02	0.79 (0.03)	0.74–0.85
Body mass index*	25.34 (0.12)	25.11–25.58	26.94 (0.20)	26.53–27.34	25.54 (0.11)	25.32–25.75

	**% (SE)**	**95% CI**	**% (SE)**	**95% CI**	**% (SE)**	**95% CI**

Gender
Male	47.82 (0.01)	45.12–50.52	43.18 (0.02)	38.79–47.69	47.26 (0.01)	44.86–49.68
Female	52.18 (0.01)	49.48–54.88	56.82 (0.02)	52.31–61.21	52.74 (0.01)	50.32–55.14
Self-rate health*
Good–excellent	85.97 (0.01)	84.15–87.60	78.38 (0.02)	74.68–81.68	85.06 (0.01)	83.33–86.64
Poor–fair	14.03 (0.01)	12.40–15.85	21.62 (0.02)	18.32–25.32	14.94 (0.01)	13.36–16.67
Depressive symptoms*
CES-D ≤ mean + SD	88.43 (0.01)	86.36–90.22	78.57 (0.02)	74.89–81.85	87.25 (0.01)	85.32–88.95
CES-D > mean + SD	11.57 (0.01)	9.78–13.64	21.43 (0.02)	18.15–25.11	12.75 (0.01)	11.05–14.68

*P < 0.05 for all comparisons between Blacks and Whites.

*CES-D, Center for Epidemiologic Studies Depression Scale*.

Table 2 presents the results of five Cox proportional hazards models for the pooled sample. High CES-D score at baseline was associated with higher risk of all-cause mortality in Model 1 (HR = 1.50, 95% CI = 1.23–1.83). Similar findings were found in Model 2 (HR = 1.31, 95% CI = 1.07–1.60). In Model 3, higher CES-D at baseline did not predict all-cause mortality (HR = 1.12, 95% CI = 0.91–1.37). In the last model, we found an interaction between race and baseline depressive symptoms on all-cause mortality (HR = 1.66, 95% CI = 1.19–2.31), suggesting a stronger effect of baseline depressive symptoms on long-term risk of all-cause mortality among Whites compared to Blacks.

**Table 2:** **Association between baseline high depressive symptoms and all-cause mortality using Cox proportional hazards models in the pooled sample**.

	Model 1		Model 2		Model 3		Model 4	
	HR (SE)	95% CI	HR (SE)	95% CI	HR (SE)	95% CI	HR (SE)	95% CI
High CES-D	1.50 (0.15)***	1.23–1.83	1.31 (0.13)**	1.07–1.60	1.12 (0.11)	0.91–1.37	1.23 (0.14)^#^	0.98–1.55
Age	1.09 (0.00)***	1.09–1.10	1.09 (0.00)***	1.08–1.09	1.08 (0.00)***	1.08–1.09	1.08 (0.00)***	1.08–1.09
Female	0.59 (0.04)***	0.52–0.67	0.55 (0.04)***	0.48–0.63	0.54 (0.04)***	0.47–0.62	0.54 (0.04)***	0.47–0.62
Education	–	–	0.97 (0.01)*	0.95–1.00	0.99 (0.01)	0.96–1.01	0.99 (0.01)	0.96–1.01
Income	–	–	0.92 (0.01)***	0.89–0.94	0.93 (0.01)***	0.90–0.96	0.93 (0.01)***	0.90–0.96
CMC	–	–	–	–	1.16 (0.03)***	1.10–1.21	1.16 (0.03)***	1.10–1.22
BMI	–	–	–	–	0.99 (0.01)	0.98–1.01	0.99 (0.01)	0.98–1.01
SRH (poor/fair)	–	–	–	–	1.52 (0.11)***	1.31–1.77	1.51 (0.11)***	1.30–1.76
Black	1.36 (0.09)***	1.19–1.55	1.15 (0.09)^#^	0.98–1.35	1.10 (0.09)	0.94–1.30	0.73 (0.11)*	0.54–0.99
High CES-D* Black	–	–	–	–	–	–	1.66 (0.27)**	1.19–2.31

*BMI, body mass index; SRH, self-rated health; CMC, chronic medical conditions; CES-D, Center for Epidemiologic Studies Depression*.

*^#^*P* < 0.1*.

*P < 0.05.

**P < 0.01.

***P < 0.001.

Table 3 presents the results of Cox proportional hazards models specific to race groups. Among Whites, in Models 1 and 2, baseline CES-D was a predictor of all-cause mortality. In Model 3, baseline CES-D did not remain as a predictor of all-cause mortality. Among Blacks, in Model 1, there was no significant association between high depressive symptoms at baseline and risk of all-cause mortality. In Model 2, the association between high depressive symptoms at baseline and mortality became marginally significant. In Model 3, higher CES-D at baseline was associated with a lower risk of subsequent mortality after controlling for all covariates, including CMC, BMI, and SRH.

**Table 3:** **Association between baseline high depressive symptoms and all-cause mortality using Cox proportional hazards models among Whites and Blacks**.

	Model 1		Model 2		Model 3	
	HR (SE)	95% CI	HR (SE)	95% CI	HR (SE)	95% CI
**Whites**
High CES-D	1.68 (0.19)***	1.34–2.12	1.46 (0.16)***	1.17–1.83	1.21 (0.14)	0.96–1.54
Age	1.10 (0.00)***	1.09–1.10	1.09 (0.00)***	1.08–1.10	1.09 (0.00)***	1.08–1.09
Female	0.57 (0.04)***	0.49–0.66	0.53 (0.04)***	0.46–0.62	0.52 (0.04)***	0.45–0.61
Education	–	–	0.96 (0.01)*	0.94–0.99	0.98 (0.01)	0.95–1.01
Income	–	–	0.92 (0.01)***	0.89–0.95	0.93 (0.02)***	0.90–0.97
CMC	–	–	–	–	1.17 (0.03)***	1.11–1.23
BMI	–	–	–	–	0.99 (0.01)	0.97–1.01
SRH (poor/fair)	–	–	–	–	1.61 (0.13)***	1.36–1.89
**Blacks**
High CES-D	0.92 (0.10)	0.75–1.14	0.82 (0.09)^#^	0.65–1.03	0.77 (0.09)*	0.61–0.97
Age	1.08 (0.00)***	1.07–1.09	1.07 (0.00)***	1.06–1.08	1.06 (0.01)***	1.05–1.07
Female	0.75 (0.08)**	0.61–0.93	0.66 (0.07)***	0.53–0.81	0.64 (0.07)***	0.51–0.81
Education	–	–	0.99 (0.01)	0.96–1.02	1.00 (0.01)	0.97–1.03
Income	–	–	0.90 (0.03)***	0.85–0.96	0.91 (0.03)**	0.85–0.96
CMC	–	–	–	–	1.12 (0.06)*	1.01–1.25
BMI	–	–	–	–	1.00 (0.01)	0.98–1.02
SRH (poor/fair)	–	–	–	–	1.13 (0.11)	0.93–1.37

*BMI, body mass index; SRH, self-rated health; CMC, chronic medical conditions; CES-D, Center for Epidemiologic Studies Depression*.

*^#^*P* < 0.1*.

*P < 0.05.

**P < 0.01.

***P < 0.001.

## Discussion

The current study had three main findings: first, race and baseline depressive symptoms interacted on all-cause mortality, suggesting a stronger effect for Whites compared to Blacks. Second, the stronger effect of baseline depressive symptoms on subsequent risk of all-cause mortality among Whites was not due to racial disparities in SES or health (i.e., CMC, SRH, and BMI). Third, health has different roles on the link between baseline depressive symptoms and long-term risk of all-cause mortality among Whites and Blacks. Although CMC suppresses the effects of depressive symptoms on mortality among Blacks, SRH explains (confounds) the same link among Whites.

Our first findings on the stronger predictive role of baseline depressive symptoms on all-cause mortality for Whites compared to Blacks are in line with a growing body of evidence, suggesting that depressive symptoms, SRH, and hostility may have a weaker predictive role for long-term mortality of Blacks compared to Whites (6, 18–21). The same findings have been shown for different predictors, different populations, and different causes of mortality (18–21). We still do not know whether these racial differences in the predictive role of baseline characteristics on long-term risk of mortality are due to different validity or measurement errors of predictors at baseline or the unpredictability or competing risks of long-term mortality among Blacks. There is also some evidence suggesting that race and ethnicity may interfere with the exact meaning of health/reflection of SRH and depressive symptoms and other health indicators (9, 17).

A recent study found that baseline depressive symptoms predict subsequent risk of major depressive disorder (MDD) 15 years later among Whites but not Blacks, suggesting a weaker effect of depressive symptoms on subsequent all-cause mortality among Blacks (54). This finding is also supported by the Black–White paradox, defined as less frequent depression despite higher prevalence of chronic medical conditions among Blacks compared to Whites in the U.S. (10, 11). Based on this paradox, we expect weaker correlations between depression and physical health (e.g., mortality) among Blacks than Whites (9, 15).

The unexpected protective residual effect of depressive symptoms on all-cause mortality over SES and health for Blacks is not easy to explain. Nesse has discussed an evolutionary explanatory model of depression. In this model, depression and lack of motivation may be a healthy reaction and may increase an organism’s ability to cope with the adaptive challenges characteristic of unpropitious situations (55). Some benefits of depression may be secondary to the role of depression such as communicating a need for help, signaling yielding in a hierarchy conflict, or fostering disengagement for commitments to unreachable goals (56). In this view, depression may promote escape and avoidance of discriminatory and racist situations in a life full of economic and other structural barriers (55). As Beck argued, depression may have an adaptive role in conserving resources in a resource-limited environment (57). Depression may be a communication tool designed to manipulate others into providing emotional and tangible support and resources (58, 59). As Lewis suggested, depression in adults is a plea for help from their close social network (60). Wolpert suggested that not clinical depression but subclinical levels are adaptive (61).

Our study showed suppressor effects of health status on the association between depressive symptoms and mortality among Blacks, which means controlling for CMC, BMI, and SRH is required if we want to study variability of mortality in Blacks due to depressive symptoms. Results suggest that researchers should decompose variance of mortality of Blacks attributable to health-related variables (i.e., CMC) from what might be explained by depressive symptoms (46). Other examples of suppressor effects exist in the literature (10, 47). However, in Whites the mortality risk associated with depressive symptoms was explained by health status.

Race and ethnicity alter the additive effects of depression/depressive symptoms and CMC on mortality (6, 9, 13, 14). Literature has shown some mixed results considering Black–White differences in the depression – CMC link. The mechanism behind lower rates of depression despite higher rates of CMC in Blacks compared to Whites remains unexplained (9, 15). Recently, a longitudinal study showed that baseline depressive symptoms predict an increase in CMC among Whites but not Blacks (9). Racial differences in the effects of CMCs (e.g., diabetes) on mortality have also been shown (37). Lower mortality rates of Blacks with diabetes compared to Whites with diabetes are very difficult to explain, particularly considering lower access of Blacks to treatment, SES, and disease management and higher comorbidities among Blacks who suffer CMC compared to Whites (37).

We found that in the pooled sample and also among Whites but not Blacks, CMC and SRH explained the association between baseline depressive symptoms and all-cause mortality. Houle showed that CMC may mediate the effect of depressive symptoms to all-cause mortality (5). Among Blacks, we found that CMC may suppress (not mediate) the effect of depressive symptoms on all-cause mortality (46). We showed that the role of CMC on the abovementioned link may vary based on race; however, Houle applied his models in the pooled sample and did not explore group differences (5).

We also found that baseline SRH explained (confounding) the effect of depressive symptoms on mortality for Whites but not Blacks. Although SRH may reflect different aspects of health across populations, the SRH-depression link may also vary based on race and ethnicity. It is also shown that race modifies the relationship between SRH and mortality (17–19, 48, 49, 62, 63). Although McGee et al. showed that SRH is associated with increased risk of mortality for all racial/ethnic groups (48), another study showed that SES alters the association between SRH and mortality (64).

There have been some studies investigating the predictive role of depressive symptoms on subsequent mortality. Houle, Atlantis et al., and White et al. showed a mediating effect of CMC on CES-D to all-cause mortality (5, 22, 65). However, none of these studies explored the role of race as a possible effect modifier. Lewis et al. explored the role of race on the link between depressive symptoms and specific cause mortality in the Chicago Health and Aging Project study (13) and found that CES-D levels predicted cardiovascular disease mortality and stroke mortality in Blacks but not Whites (13). However, Capistrant et al. could not replicate findings reported by Lewis et al. using data of the Health and Retirement study (14).

Our findings have implications for racial health disparities in the U.S., which have existed for several decades, particularly a lower life expectancy for Blacks (66–68). The differences in depression and the effects of health on mortality in Whites and Blacks suggest that SES, CMC, SRH, and depressive symptoms may be differently important for life expectancy of Whites and Blacks in the U.S., which should be taken into account by the health policy makers. Further scrutiny is warranted to determine the exact burden of the issue.

As our study is subject to a number of limitations, the results should be interpreted with caution. First, we used the baseline depressive symptoms, CMC, SRH, and BMI; however, all these conditions are dynamic and subject to change over time. Second, we did not control for the effect of baseline MDD diagnosed by a clinician. Third, we could not control for the use of antidepressants by the participants. Fourth, we did not control for other potential covariates, including daily activity or access to medical care. We also did not check for a potential interaction of depressive symptoms with gender and age, considering that older adults were oversampled in ACL study and depressive symptoms are more prevalent in women than men. Fifth, we used a self-reported measure of CMC. Although high validity of self-rated medical conditions is known (69, 70), future research will benefit from using medical record information. Finally, our sample size of Blacks with high depressive symptoms was small. Most of the mentioned limitations have existed in the previous studies investigating depressive symptoms and mortality (5, 13, 14, 22). Despite these limitations, according to our knowledge, this is one of the first studies to investigate the moderating role of race on the residual effect of depressive symptoms on all-cause mortality, over SES, and health. One strength of our study is that our results are generalizable to the U.S. population because we used 25 year follow up data of more than 3,000 Americans representative of the U.S. population. Using race stratified models to further explore the direction and magnitude of the interaction in the pooled sample and using a hierarchical approach for data analysis that provides a comprehensive understanding about race differences in the associations is also a strength.

Overall, our findings are suggestive of a moderating effect of race on residual effects of baseline depressive symptoms over SES and health on subsequent risk of all-cause mortality over a 25-year follow-up period in the U.S. Further research is needed to better understand the exact mechanisms behind Black–White differences in the complex links between SES, physical health, and mental health on all-cause mortality in the U.S.

## Informed Consent

Informed consent was obtained from all participants included in the study.

## Ethics

All procedures followed were in accordance with the ethical standards of the responsible committee on human experimentation (institutional and national) with the Helsinki Declaration of 1975, as revised in 2000. University of Michigan Institutional review board (IRB) approved the study protocol.

## Author Contributions

SA designed and analyzed this work, and contributed to draft and revision. EM-Z made an extensive literature review, drafted and revised the manuscript. ML and VM-F provided contribution to the first draft and also revisions. All authors confirmed the last version.

## Conflict of Interest Statement

The authors declare that the research was conducted in the absence of any commercial or financial relationships that could be construed as a potential conflict of interest.
